# Decontamination and Reuse of N95 Masks: A Narrative Review

**DOI:** 10.1155/2020/8869472

**Published:** 2020-11-25

**Authors:** M. A. Khan, A. Ikram, S. Savul, F. K. Lalani, M. A. Khan, M. Sarfraz

**Affiliations:** National Institute of Health, Islamabad, Pakistan

## Abstract

**Background:**

The COVID-19 pandemic has presented an unprecedented strain on healthcare supplies. Currently there is a global shortage of personal protective equipment (PPE), especially N95 masks. In order to safeguard healthcare personnel in this critical time and to mitigate shortages of N95 respirators, reuse of N95 respirators has to be considered.

**Methods:**

Using PubMed and Science Direct, a literature search was conducted to find and synthesize relevant literature on decontamination of N95 respirators for their subsequent reuse. Peer-reviewed publications related to methods of decontamination from January 2007 to April 2020 in the English language are included in this narrative review. Bibliographies of articles for relevant literature were also scrutinized. *Findings*. A total of 19 studies are included in this narrative review. The appraised methods include ultraviolet germicidal irradiation (UVGI), moist heat incubation (MHI), ethylene oxide (EtO), hydrogen peroxide vapor (HPV), microwave steam bags (MSB), microwave-generated steam (MGS), dry microwave oven irradiation, hydrogen peroxide gas plasma (HPGP), dry heat, liquid hydrogen peroxide, and bleach and alcohol.

**Conclusion:**

In light of the COVID-19 pandemic, reuse of N95 respirators, although suboptimal, can be considered. Evidence reveals that UVGI, MHI, and HPV are amongst the safest and efficacious methods for decontamination of N95 masks. More research is needed to establish the safety and effectiveness of MGS, MSB, dry heat, EtO, liquid hydrogen peroxide, and HPGP. Alcohol, microwave irradiation, and bleach are not recommended because they damage N95 respirators.

## 1. Background

The COVID-19 pandemic caused by SARS-CoV-2 virus has created a shortage of PPE globally. Keeping in view the nature of transmission of the virus and its contagiousness, the demand of N95 respirators has increased drastically. Nosocomial transmission of the virus has also been reported, and substantial numbers of healthcare workers have been affected worldwide [1]. N95 respirators provide essential protection against the virus owing to of their tight fit and efficient filtration capacity [2].

In general, N95 respirators are designed for single use prior to disposal. They must be discarded after performing aerosol generating procedures, when damaged, deformed, visibly dirty or when they stop forming an effective seal on the face [3]. The rationale for these recommendations may be due to the fact that respiratory viruses can remain viable on respirators for an extended period of time [4, 5]. SARS-CoV-2, although more stable on plastic and stainless steel than on copper and cardboard, can remain viable for up to 72 hours [6]. Extended use of N95 respirators implies using the same respirator for an extended period of time with donning and doffing carried out only once. This was previously endorsed during pandemic situations with respiratory pathogens to mitigate shortage of respirators [7, 8]. Although extended use of respirators may bring about physical discomfort to the healthcare workers, it is endurable without any additional health risks [9].

In order to deal with the gap in the demand and supply of N95 respirators, guidelines proposed by the Infectious Diseases Society of America now recommend optimization of personal protective equipment [10]. In order to mitigate shortage of respirators, reuse after decontamination has been recommended which is based on laboratory evidence as there is lack of clinical experience [10, 11].

A range of decontamination methods for N95 respirators have been studied. Various characteristics of the respirator have to be taken into account including modifications in their physical appearance/odor, structural integrity, filtration efficiency, fit, seal and airflow resistance, and degradation of the material and chemical residues that may be irritant to the skin. The equipment needed for decontamination, its ease of use, cost, and the allowed number of decontamination cycles must also be considered [3]. This article aims to present a review of different methods used for decontamination of N95 respirators for their subsequent reuse.

## 2. Methods

A literature search was conducted using PubMed and Science Direct. The following search terms were used: “Decontamination”(Mesh) OR “Reuse”(Mesh) OR “Disinfection”(Mesh) AND “Respiratory Protective Devices”(Mesh) OR ″”N95 Respirator (Mesh)” OR “Filtering Facepiece Respirator (Mesh).” Peer-reviewed publications related to methods of decontamination of N95 respirators and their reuse from January 2007 to April 2020 in the English language are included in this review. Bibliographies of articles for relevant literature were also scrutinized. Because this is a narrative review, all relevant studies were included regardless of the sample size. Non-English language publications and those for which full text was not available were not eligible for inclusion in the review.

## 3. Results

A total of 192 studies were found via electronic search. 168 studies were not deemed relevant after scrutinizing the title, abstract, and then the full text and hence were excluded from the review. Five additional studies were identified after examining the reference lists of relevant articles. A total of 19 studies are presented in the review classified by the method of decontamination. The appraised methods include ultraviolet germicidal irradiation (UVGI), moist heat incubation (MHI), ethylene oxide (EtO), hydrogen peroxide vapor (HPV), microwave steam bags (MSB), microwave-generated steam (MGS), dry microwave oven irradiation, hydrogen peroxide gas plasma (HPGP), dry heat/hot air oven, liquid hydrogen peroxide, and bleach and alcohol. Some studies assessed multiple methods of decontamination, but they are counted as one publication although the results are presented separately in Table 1.

## 4. Discussion

### 4.1. Ultraviolet Germicidal Irradiation (UVGI)

Ultraviolet germicidal irradiation (UVGI) is a disinfection method that employs short-wavelength ultraviolet *C* or UV-C light to destroy microorganisms. [31]. UVGI has been successfully used to disinfect medical equipment [32] and high touch surfaces in hospital [33]. Several studies have studied the effects of decontamination of N95 respirators using UVGI, and the majority of them evaluated the performance of a single cycle (15 minutes) of UVGI on N95 respirators. However, two studies evaluated the performance of three cycles of UVGI [19, 23]. A varying range of one to fifteen N95 respirators were employed in these studies. Five studies assessed particle penetration posttreatment with UVGI [14, 15, 17, 19, 23]. Apart from Lindsley et al. [14] whose study took into account five UVGI intervention arms, the rest evaluated a single UVGI protocol. Particle penetration for masks ranged from 0.7% to 2.48%. For the control group, particle penetration ranged from 0.57% to 2.63%. Fisher et al. [22] described aerosol penetration for the individual layers of six N95 FFR models and found that the majority of filter layers had an efficiency of >94%. In studies that assessed airflow resistance, average values ranged from 7.98 mm to 11.44 mm H_2_O for the control and post-UVGI treatment arms [14, 15, 19]. However, Fisher et al. reported the airflow resistance of the individual filtering layers between 2.5 mm and 7.6 mm H_2_O [22].

Evidence demonstrates that the germicidal impact of UVGI is good [12, 16, 20–22, 24] with majority of studies examining its effect on H1N1 and MS2. In only one study, a *Bacillus subtilis* prototype strain was used [21]. Multiple studies assessed physical changes in respirators following UVGI treatment by visual inspection [15, 17–19, 23]. No significant changes in physical appearance have been noted. No significant changes in odor were observed in the studies that evaluated this aspect [18, 19]. In two studies assessing fit of the respirator, no significant reduction in fit was found [18, 23]. In view of the available evidence, UVGI is an effective method of decontamination of N95 respirators and is endorsed by the Food and Drug Administration (FDA) [3].

### 4.2. Ethylene Oxide (EtO)

Ethylene oxide (EtO) is a commonly used sterilizing agent for medical equipment [34]. However, CDC advocates using ethylene oxide with caution for sterilizing N95 masks, as it is carcinogenic and teratogenic and can cause neurologic dysfunction thus harming the wearer [3]. Salter et al. [25] measured the amount of residual chemicals created or deposited on N95 masks with EtO treatment and reported that it resulted in detectable toxins.

A study utilizing two EtO sterilization protocols revealed that ethylene oxide did not negatively impact filtration performance for one tested N95 model [15]. Another study showed that EtO did not affect physical appearance, filter aerosol penetration, and filter airflow resistance of six models of N95 respirators [17]. Bergman et al. evaluated the physical changes, odor, and laboratory filtration performance of six models of N95 respirators after EtO treatment and reported no significant changes [19]. However, these studies [15, 17, 19] did not evaluate FFR fit testing after EtO treatment.

### 4.3. Hydrogen Peroxide Vapor (HPV)

HPV is a vapor form of hydrogen peroxide (H2O2) with cidal action against a wide range of microorganisms and nontoxic to human health [35]. Evidence delineates that HPV does not compromise the efficacy and performance of N95 respirators and can be used for simultaneous sterilization of a large number of used but intact respirators. However, it can only be utilized for certain types of N95 models, e.g., 1860 which do not contain cellulose [36].

A study carried out in 2007 by Viscusi et al. [15] utilized T1, T7, and *Pseudomonas* phage phi-6 as proxies for SARS-CoV-2 and established that HPV decontamination resulted in complete eradication of phage from one model of the N95 respirator without causing any physical damage or affecting filtration performance. In another study [27], the virucidal activity of HPV was evaluated using a BQ-50 system. In this experiment, a single HPV cycle resulted in complete eradication of phage from masks, and no physical deformity was observed after 5 cycles [27]. Half of the respirators underwent steam sterilization at 275°F for 5 min which degraded the respirators and resulted in no additional virucidal action [27].

Two studies established the efficacy of HPV against *Geobacillus stearothermophilus* spores [26, 28]. Battelle Memorial Institute reported a 6 log reduction without affecting filtration performance and noted that the performance of N95 FFRs exceeded 95% efficiency after 50 cycles and minor degradation in elastic respirator straps occurred after 30 cycles [26]. T Schwartz et al. [28] performed decontamination successfully with no physical or performance degradation of FFRs using commercially available equipment, and fit testing was conducted on humans instead of mannequins.

### 4.4. Liquid Hydrogen Peroxide

Hydrogen peroxide is a colorless liquid which can be used for disinfection of medical devices and surfaces [37]. Decontamination of N95 respirators with liquid hydrogen peroxide is an encouraging method but it has limitations. This method has been assessed by only two studies. Viscusi et al. [15] submerged samples of one model of N95 in 3% and 6% hydrogen peroxide solutions for 30 min and reported no change in filtration performance; however, 6% hydrogen peroxide slightly faded label ink on the respirator fabric. Bergman et al. [19] demonstrated that soaking six FFR models in 6% solution of hydrogen peroxide for 30 min did not affect filtration performance. However, in this study, the fit integrity was not tested, and staples oxidized to varying degrees in N95 models with staples [19].

### 4.5. Hydrogen Peroxide Gas Plasma (HPGP)

HPGP is the nontoxic method of sterilization which inactivates wide range of microorganisms by the combined use of hydrogen peroxide gas and generation of free radicals and is useful for materials that cannot endure high temperatures and humidity [38]. Conflicting results were found for the use of hydrogen peroxide gas plasma for decontamination of N95 respirators. Viscusi et al. [17] decontaminated six models of N95 FFRs using the STERRAD100S H_2_O_2_ Gas Plasma Sterilizer and found that one treatment cycle had filter aerosol penetration and filter airflow resistance levels similar to untreated models. However, Bergman et al. [19] reported that three treatment cycles using the same machine negatively affected the filtration performance of N95 respirators. Although Bergman et al. [19] reported no physical changes in respirators, Viscusi et al. [17] found that HPGP treatment tarnished metallic nosebands of respirators with loss of shine.

### 4.6. Hot Air Oven/Dry Heat

Hot air oven is a form of dry heat sterilization in which microorganisms are destroyed by exposing them to extremely high temperatures over several hours [39]. Limited evidence is available for dry heat decontamination, and the optimal parameters for temperature and duration are ambiguous. A few studies have shown encouraging results. Liao et al. [13] kept respirators inside a hot air oven at 70°C for 30 min, demonstrating favorable results in terms of fit and filtration efficiency. Fischer et al. [29] exposed contaminated respirators to 70°C heat in an oven. Quantitative fit tests showed that the filtration performance of the N95 respirator was retained after decontamination and respirators may be utilized up to two times [29].

### 4.7. Moist Heat Incubation (MHI)

Moist heat incubation is usually performed between 60% and 85% relative humidity (RH) in laboratory incubators where warm moisture causes biocidal action. Moist heat is more effective than dry heat in destroying microorganisms and less likely to negatively impact filter performance [15, 17]. Data from multiple studies revealed encouraging results for the decontamination and reuse of N95 respirators using MHI. However, there is limited available evidence on the ability of MHI to disinfect different pathogens [3]. Heimbuch et al. [20] used moist heat to sterilize H1N1-contaminated N95 masks and demonstrated 99.99% reduction in H1N1 influenza virus. Lore et al. [16] found that moist heat effectively decontaminated two N95 models contaminated with H5N1. Bergman et al. [19] performed a 30-minute incubation of six models of N95 at 60°C with 80% relative humidity and ascertained minimal changes in physical appearance, odor, and filtration performance of the masks. Both Lore et al. [16] and Bergman et al. [19] found the post-decontamination filtration efficiency to be greater than 97.5% [16, 19]. Viscusi et al. [18] reported that decontamination of N95 respirators with MHI did not cause significant reduction in fit, increase in odor, increase in discomfort, or increased difficulty in donning, thereby rendering it a safe and effective method. The exposure time per decontamination cycle was 20 minutes in one study [16] and 30 minutes in three studies [18–20]. Another study [23] utilized a shorter decontamination cycle of 15 min and reported that MHI did not negatively impact fit and filtration efficiency.

### 4.8. Microwave Steam Bags (MSB)

Commercially available microwave steam bags (MSBs) are available in retail stores and are commonly used to decontaminate breast infant feeding accessories [30]. A study by Fischer et al. [30] employed two steam bags for decontamination of N95 respirators and tested them for water absorption and filtration efficiency. Steam exposure was given thrice. Efficacy was evaluated using bacteriophage MS2 as a surrogate for the pathogenic virus. The tested steam bags were found to be 99.9% effective for inactivating MS2 on FFRs and filtration efficiency remained above 95% [30]. However, further research is needed to establish the effectiveness of this method.

### 4.9. Microwave-Generated Steam (MGS)

Microwaves are commonly available electrical appliances, and several studies have revealed beneficial decontamination effects for microwave-generated steam. However, inadequate controls for uniform steam distribution, the unknown effect of microwave power capacity on respirators, and danger of combustion of FFR metal nosebands must be taken into account [34]. Two studies reported nonsignificant effects on filtration and fit performance of N95 respirators employing one decontamination cycle [16, 20]. Using MGS, Heimbuch et al. [20] revealed a minimum of 99.9% reduction in viable H1N1 virus, and Lore et al. [16] reported 99.9% reduction in bacteriophage MS2. In a study performed by Bergman et al. [19], the filtration efficiency remained intact after three cycles of exposure to microwave-generated steam, and no sparking was observed. However, slight physical changes were seen in two N95 models [19].

### 4.10. Other Methods—Dry Microwave Oven Irradiation

This method is not recommended for decontamination of FFRs due to significant filter degradation and damage to masks [40]. Viscusi et al. [17] exposed six models of N95 FFRs for 2 minutes at maximum power setting in a commercially available 2450 MHz microwave oven. After treatment, the SN95-E model melted in areas adjacent to the metallic nosebands and therefore was not assessed further for filter aerosol penetration or filter airflow resistance [17]. In other models, filter aerosol penetration and filter airflow resistance were unaffected [17].

### 4.11. Other Liquid Disinfectants—Bleach and Alcohol

Bleach (sodium hypochlorite) and alcohol are not recommended for decontamination of N95 respirators. According to the World Health Organization and Centres for Disease Control and Prevention, bleach and alcohol cause damage to N95 masks, loss of filtration efficiency, and have potential for toxicity [3, 40]. A recent study [13] reported that alcohol and chlorine-based solutions are detrimental to the static charge in the microfibers of N95 masks and result in reduced efficiency. In addition, chlorine-based solutions generate odor and harmful fumes after decontamination [13, 15, 17].

## 5. Conclusion

Due to the existent shortage of PPE in the current COVID-19 pandemic, reuse of N95 respirators may be considered, although it has limitations since respirators are inherently designed to be single use items. In light of the above evidence, ultraviolet germicidal irradiation, moist heat incubation, and vaporous hydrogen peroxide are the most efficacious and safe methods for decontamination of N95 respirators. Microwave-generated steam, microwave steam bags, dry heat, ethylene oxide, liquid hydrogen peroxide, and hydrogen peroxide gas plasma are promising methods but the amount of available evidence is limited to recommend them as first line methods. Alcohol, microwave irradiation, and bleach are not recommended because they can damage N95 respirators and cause filter degradation.

The safety and effectiveness of N95 respirators after decontamination in clinical settings still remains a question. It is suggested that studies proposing methods of decontamination must ensure maximal decontamination (reduction in viral load) as well as preservation of the integrity, and filtering function and proper seal of the mask. It is envisioned that high quality experiments designed to address the decontamination of N95 respirators with COVID-19 in real clinical settings will shed light on these issues in the future.

## Figures and Tables

**Table 1:** Methods of decontamination.

Methodology	Key results	Study
UVGI		
15 N95 FFR models (3M 1860, 3M1870, 3M VFlex 1805, Alpha Protech 695, Gerson 1730, Kimberly-Clark PFR, Moldex 1512, Moldex 1712, Moldex EZ-22, Precept 65–3395, Prestige Ameritech RP88020, Sperian HC-NB095, Sperian HC-NB295, U.S Safety AD2N95 A, and U.S Safety AD4N95) contaminated with H1N1 inﬂuenza and covered with a soiling agent (artiﬁcial saliva or artiﬁcial skin oil) were placed in a custom UV device made of polished aluminum and irradiated with UV dose of 1 J/cm^2^ in ∼1 min.	Signiﬁcant reductions (≥3 log) in inﬂuenza viability for both soiling conditions were observed on facepieces of 12 of 15 FFR models and straps from 7 of 15 FFR models.	Mills et al., 2018 [12]
3 N95 models of N95 were placed into a UV sterilizer cabinet (CHS-208A), with a 254 nm, 8 W lamp, and 475 cm^2^ internal area. Samples were irradiated for 30 minutes and left to stand under ambient conditions for 10 minutes per cycle for 10 cycles.	After 10 cycles of UV treatment, the meltblown filtration efficiency and pressure drop remained constant.	Liao et al., 2020 [13]
4 models of N95 (3M 1860, 3M 9210, Gerson 1730, and Kimberly-Clark 46727) were exposed to UVGI doses (UV-C) from 120 j/cm^2^ to 950 j/cm^2^ in a custom-made 91 cm × 31 cm × 64 cm high chamber.	UVGI exposure led to a small increase in particle penetration (up to 1.25%) and a little effect on the ﬂow resistance. Some degradation of elastic straps was noted on exposure to higher UV levels	Lindsley et al., 2015 [14]
1 model of N95 was placed on the working surface of a laminar flow hood, Sterilgard III, (The Baker Company, Sanford, ME) fitted with a 40 W ultraviolet light.	No visible changes were observed. Various parts, e.g., straps, nose clips, and exhalation valves remained intact. Average penetration was not altered significantly. Filtration efficiency was good.	Viscusi et al., 2007 [15]
Using 126 (*L*) % 15.2 (W) % 10.8 cm (H), dual-bulb, 15-W UV-C (254 nm wavelength) lamp (Ultraviolet Products, Upland, CA, USA) was placed in a Labgard class II, type A2, laminar flow cabinet (NuAire, Inc., Plymouth, MN, USA), 3M 1860 and 3M 1870 were decontaminated subjected to treatment for 15 min at a UV-C wavelength dose of 18 kJ m^2^.	Reduction of virus load by > 4 log median tissue culture infective dose (>10,000-fold reduction in H5N1) for both FFRs was observed.	Lore et al., 2012 [16]
6 N95 FFRs (N95-A, N95-B, and N95-C, SN95-D, SN95-E, and SN95-F) were placed on the working surface of a Sterilgard III laminar flow cabinet (The Baker Company, Sanford, ME) fitted with a 40-WUV-C light with 176–181 mJ cm^−2^ exposure to each side of the FFR, for a total time of 30 minutes.	UVGI treatment did not affect filtration aerosol penetration, filter airflow resistance, and physical appearance of FFRs.	Viscusi, et al. 2009 [17]
FFRs (3M 8000, 3M 8210, Moldex 2200, 3M 1860, 3M 1870, and Kimberly-Clark PFR95–270) were placed in a Sterilgard III laminar flow cabinet (The Baker Company, Sanford, Maine) fitted with a 40W UV-C bulb, intensity 1.8 mW/cm^2^ measured with a UVX Digital Radiometer with a MODEL UVX-25 sensor (254 nm filter). The total exposure time was 30 min (15 min each FFR side).	No significant changes in fit, comfort, or donning difficulty were noted.	Viscusi, et al., 2011 [18]
6 models of N95 (N95-A, N95-B, and N95-C and SN95-D, SN95-E, and SN95-F) were decontaminated using a UV bench lamp (UV-C, 254 nm, 40 W), Model XX-40S (UVP, LLC, Upland, CA). 45 minute exposure at intensity 1.8 mW/cm^2^ was given.	All models had expected levels of both filter aerosol penetration and filter airflow resistance with no visible physical changes.	Bergman et al., 2010 [19]
15 N95 FFR models (3M 1870, 3M 1860, Kimberly-Clark PFR, Moldex 1512, Precept 65–3395, Gerson 1730, Sperian HC-NB 095, U.S Safety AD2 N95 A, Moldex 1712, US Safety 4 N95, 3M VFlex 1805, Alpha Protech 695, Prestige Ameritech RP 88020, Sperian HC-NB295 F and Moldex EZ-22) were placed 25 cm below of a 120 cm, 80 Watts UV-C lamp (Mineralight XX-20S 20-W UV bench lamp) for a total of 15 min exposure.	Average log reduction of 4.69, virus reduced to values below the detection limit. No significant physical changes or changes in odor were observed.	Heimbuch et al., 2011 [20]
The relative survival of *Bacillus subtilis* spores was determined by loading onto N95 models (8210, 3M, St. Paul, and MN) after UVGI (UVA 365 nm and UVC 254 nm) decontamination under 37°C and relative humidity of 95%. FFRs was placed 10 cm below a 6 W handheld UV lamp (model UVGL-58, VUP LLC, Upland, CA) that emitted a wavelength of 254 nm (UV-C, 18.9 mW/cm^2^) or 365 nm (UVA, 31.2 mW/cm^2^)	No colony was recovered after exposure to UV-C for as little as 5 minutes. Relative survival remained above 20% after 20 minutes of irradiation by UVA, exponentially decaying with increased exposure time.	Lin et al., 2018 [21]
N95 respirators (8210, Cardinal N95-ML, Wilson SAF-T-FIT, 3M 1860, 3M 1870, and Kimberly-Clark PFR95–174) exposed to aerosolized particles containing MS2 coliphage were treated with internal filtering medium- (IFM-) specific UV-C doses ranging from 38 J m^−2^ to 4707 J m^−2^ using a biological safety cabinet (SterilGARD® III Advance; The Baker Company, Sanford, ME, USA).	Models exposed to a minimum IFM dose of 1000 J m−2 demonstrated at least a 3 log reduction in viable MS2. Model‐specific exposure times to achieve this IFM dose ranged from 2 to 266 min.	Fisher et al., 2010 [22]
3 models of N95 respirators (3M 1860, 3M 1870, and Kimberly-Clark PFR95-270 (46767) were placed on a laboratory stand inside a Sterilgard III laminar flow cabinet (The Baker Company, Sanford, ME) fitted with a 40 W UV-C bulb for 15 minutes.	Respirator fit was preserved through 3 decontamination cycles alternating with 4 donning/doffing cycles. No physical degradation of FFRs was observed.	Bergman et al., 2011 [23]
N95 FFR (model N1105) loaded with MS2 coliphage was decontaminated by a UV germicidal lamp in a biological safety cabinet (SterilGARD III model SG403 A; Baker Company, Sanford, ME) using low doses of 4.32–5.76 J/cm^2^ and high doses ≥ 7.20 J/cm^2^ with a 5-hour irradiation time.	Low UVGI dose resulted in 3.00- to 3.16-log reductions, and high doses resulted in no detectable MS2 virus.	Vo et al., 2009 [24]
Ethylene oxide (EtO)		
One model of N95 subjected to two treatments: EtO 3M Steri-Vac 4XL sterilizer at 55°C and 883 mg/L ethylene oxide gas and EtO 3M Steri-Vac 5XL sterilizer at 55°C and 725 mg/L ethylene oxide gas. All samples underwent 1 h exposure followed by 4 h aeration.	No negative effect on filtration performance. Average penetration of masks was slightly increased but not beyond their respective NIOSH certification criteria.	Viscusi et al., 2007 [15]
Steri-Vac 5XL 100% ethylene oxide sterilizer at 55°C; 1 h exposure (725 mg/L) followed by 4 h aeration. Six models of N95 (N95-A, N95-B, N95-C, SN95-D, SN95-E, and SN95-F) were evaluated.	EtO treatment did not affect the filter aerosol penetration, filter airflow resistance, or physical appearance of FFRs.	Viscusi et al., 2009 [17]
Amsco Eagle 3017 100% ethylene oxide sterilizer at 55°C; 1 h exposure (736.4 mg/L) followed by 12-h aeration. Six models of N95 (N95-A, N95-B, N95-C, SN95-D, SN95-E, and SN95) were utilized.	No visible physical changes to the appearance of FFRs or effect on filtration performance.	Bergman et al., 2010 [19]
Using Amsco Eagle 3017 sterilizer, the amount of residual EtO on six models of FFRs (S1, S2, S3, P1, P2, and P3) was measured. Headspace solid-phase microextraction (HSSPME) analysis using gas chromatography-mass spectrometry (GC-MS) was performed.	No residual EtO was detected in any of the respirators or respirator components tested.	Salter et al., 2010 [25]
Hydrogen peroxide vapor (HPV)		
N95 FFRs (model 1860) inoculated with *Geobacillus stearothermophilus* spores were subjected to 50 repeated aerosol inoculation/decontamination cycles using a Bioquell Clarus *C* HPV generator. The 8-hour HPV cycle included a 10 min conditioning phase, 20 min gassing phase at 2 g/min, 150 min dwell phase at 0.5 g/min, and 300 min of aeration.	Decontamination of the respirator can be carried out for up to 50 cycles with >95% efficiency. After 30 HPV cycles, the elastic material in the straps split when stretched affecting respirator fit.	Battelle, 2016 [26]
Clarus *C* system (Bioquell, Horsham, PA), 3M 1870 N95 respirators (3M, St. Paul, MN) with 3 aerosolized bacteriophages; T1, T7, and *Pseudomonas* phage phi-6. Sterilization was performed with BQ-50 using a 10 min conditioning phase, 30–40 min gassing phase (varies with humidity and room size) at 16 g/min, 25 min dwell phase, and a 150 min aeration phase.	Single cycle resulted in complete eradication of phage from masks. After 5 cycles, the respirators appeared similar to new with no deformity.	Viscusi et al., 2007 [15]
3M 1870 N95 respirators inoculated with 3 aerosolized bacteriophages (T1, T7, and *Pseudomonas* phage phi-6) were suspended by their elastic on racks in 33 m^3^ room and sterilized with the BQ-50 system (Bioquell, Horsham, PA) using the 10 min conditioning phase, 30–40 min gassing phase at 16 g/min, 25 min dwell phase, and a 150 min aeration phase. Half of the respirators underwent steam sterilization at 275°F for 5 min.	Single HPV cycle resulted in complete eradication of phage from masks. After 5 HPV cycles, the respirators had no deformity. Steam sterilization degraded the respirators with no additional virucidal activity.	Kenney et al., 2020 [27]
3M1860 N95 FFRs loaded with *Geobacillus stearothermophilus* spores were decontaminated using Bioquell *Z* vaporizer (Andover, United Kingdom) utilizing 30% hydrogen peroxide solution programmed to gas for 20 min at 10 g/min, reaching ∼500 peak ppm followed by aeration of 4 hours.	Nonstatistical significant degradation of mask components. No physical or performance degradation and no noticeable odor.	Schwartz et al., 2020 [28]
Liquid hydrogen peroxide		
Submersion of samples of one type of N95 in a dishpan containing 3–5 liters of 3% hydrogen peroxide for 30 min and 6% hydrogen peroxide for 30 min. Respirators were hung on a pegboard and air-dried for 72 h.	Use of 3% hydrogen peroxide exhibited no visible changes. Use of 6% solution slightly faded label ink on the fabric of the respirator. Average penetration did not significantly change for the N95 respirator and remained well below the NIOSH 5% criteria.	Viscusi et al., 2007 [15]
30 min submersion of six N95 models (N95-A, N-95 B, N-95 C, SN-95 *D*, SN-95 *E*, and SN-95-f) in 6% solution of hydrogen peroxide over three cycle processing. FFRs were hung on a laboratory peg board and dried for a minimum of 16 h using a fan.	All models demonstrated no changes in filter performance after three cycles of decontamination. Staples oxidized to varying degrees in models with staples, i.e., N95-B, N95-C, SN95-E, and SN95-F.	Bergman et al., 2010 [19]
Hydrogen peroxide gas plasma (HPGP)		
Six models (N95-A, N95-B, N95-C, SN95-D, SN95-E, and SN95-F) were sterilized using STERRAD® 100S H_2_O_2_ Gas Plasma Sterilizer (Advanced Sterilization Products, Irvine, CA, USA) with a 55-minute standard cycle	Aerosol penetration and filter airflow resistance of the respirators were not significantly affected.	Viscusi et al. 2009 [17]
Six models (N95-A, N95-B, and N95-C, SN95-D, SN95-E, and SN95-F) were sterilized using STERRAD® 100S H_2_O_2_ Gas Plasma Sterilizer (Advanced Sterilization Products, Irvine, CA) with 59% H_2_O_2_ and cycle time ∼55 min at 45°C–50°C. Samples were packaged in Steris Vis-U-All low temperature Tyvek®/polypropylene–polyethylene Heat Seal Sterilization pouches.	FFR filtration and the fit test were passed. No physical changes in masks were observed. No significant changes in aerosol penetration	Bergman et al. 2010 [19]
Hot air oven		
N95 level meltblown filtration fabric and N95 respirators were kept in a hot air oven at 75°C for 30 minutes and variable humidity (up to 100% relative humidity)	50 cycles of heat treatment did not deteriorate filtration efficiency. No visible mechanical damage, and ear straps retained elasticity.	Liao et al., 2020 [13]
50 *μ*l of 105 58 TCID50/mL of SARS-CoV-2 was applied on N95 fabric and stainless steel and exposed to 70°C dry heat	Heat caused more rapid inactivation on N95 than on steel. Filtration performance was unaffected after one decontamination round but subsequent rounds significantly reduced filtration ability.	Fischer et al., 2020 [29]
Moist heat incubation (MHI)		
Six N95 models (N95-A, N95-B, N95-C, SN95-D, SN95-E, and SN95-F) were processed over 3 cycles for 30 min incubation at 60°C and 80% RH in the Caron model 6010 incubator	All models filtered >95% of 300 nm particulate after 3 cycles. Mean % is *P* < 4.01%, which is similar to penetration levels found in untreated fabric. SN95-E exhibited partial separation of the inner foam nose cushion from the FFR.	Bergman et al., 2010 [19]
Three N95 models (3M 1860, 3M 1870, and KC PFR95- 270) underwent incubation at 60°C for 15 min and 80% RH in the Caron model 6010 incubator for up to three cycles	Respirator fit was maintained throughout three cycles alternating with four donning/doffing cycles. Slight separation of the inner foam nose cushion was observed in 3M 1870. Face seal leakage value was below 1%.	Bergman et al., 2011 [23]
Six N95 models (M 8000, 3M 8210, Moldex 2200, 3M 1860, 3M 1870, and Kimberly-Clark PFR95–270) were decontaminated in a Caron model 6010 laboratory incubator (Marietta, Ohio) at 60°C for 30 min at 80% relative humidity	No significant changes in fit, odor detection, comfort, or donning difficulty were noted for any model	Viscusi et al., 2011 [18]
Six models contaminated with H1N1 influenza virus were subjected to 30 min of MHI at 65°C and 85% relative humidity	A >4-log reduction of viable H1N1 virus was noted.	Heimbuch et al., 2011 [20]
N95 FFRs (3M models 1860s and 1870) contaminated with H5N1 placed in a 6 L sealable container with 1 L of tap water was heated to 65 ± 5°C for 3 hours in an oven	Reduction of virus load by > 4 log median tissue culture infective dose (>10,000-fold reduction in H5N1). All FFRs displayed <5% penetration by 300-nm particles with no significant reduction in filtration performance.	Lore et al., 2012 [16]
Microwave steam bags (MSB)		
N95 masks (3M 1860, 3M 8210, Cardinal Health N95, 3M 1870, Kimberly-Clark PFR 95, and Moldex 2200) contaminated with bacteriophage MS2 were exposed to steam treatment thrice	Tested steam bags were found to be 99.9% effective for inactivating MS2 on FFRs. Filtration efficiency was above 95%.	Fisher et al., 2011 [30]
Microwave-generated steam (MGS)		
Two N95 models (3M 1860 and 3M 1870) contaminated with influenza (A/H5N1) virus were placed above a plastic box filled with 50 ml room temperature tap water, and the top of the box perforated with 96 holes (7 mm diameter). FFRs were irradiated for 2 min at full power in a 1250-W (2450 MHz) commercially available microwave oven (Panasonic corp., Secaucus, NJ, USA)	An absolute reduction of >4 log was achieved	Lore et al., 2012 [16]
	Mean penetration at 300 nm was <5% for both models with no significant degradation of filter performance	
Six FFR models contaminated with H1N1 influenza virus were exposed to MGS at 1250 W for 2 minutes	More than 4-log reduction of viable H1N1 virus was noted	Heimbuch et al., 2011 [20]
Six models (N95-A, N95-B, N95-C, SN95-D, SN95-E, and SN95-F) were processed in the commercially available 2450-MHz, Sharp Model R–305KS (Sharp Electronics, Mahwah, NJ) microwave oven; 750 W/ft3 experimentally measured; 2 min total exposure duration at maximum power. Two pipette tip boxes placed side-by-side were filled with 50 mL room-temperature tap water.	No change in filter performance after three cycles. SN95-E samples experienced partial separation of the inner foam nose cushion and slight melting of head straps of two SN95-Ds following the first 2 min cycle. The mean initial filter penetration was ≤4.01%.	Bergman et al., 2010 [19]
